# Knowledge of FAS and the Risks of Heavy Drinking During Pregnancy, 1985 and 1990

**Published:** 1994

**Authors:** Mary C. Dufour, Gerald D. Williams, Karen E. Campbell, Sherrie S. Aitken

**Affiliations:** Mary C. Dufour, M.D., M.P.H., is deputy director of the Division of Biometry and Epidemiology, National Institute on Alcohol Abuse and Alcoholism (NIAAA), Rockville, Maryland. Gerald D. Williams, D.Ed., is a senior associate at CSR, Incorported, and a subcontract manager on NIAAA’s Alcohol Epidemiologic Data System (AEDS), which is operated by Cygnus Corporation, Washington, DC. Karen E. Campbell, M.S., is a research analyst at CSR, Incorporated, Washington, DC, and is assigned to the AEDS subcontract. Sherrie S. Aitken, Ph.D., is project monitor of the AEDS subcontract and president of CSR, Incorporated, Washington, DC

## Abstract

Reducing the incidence of FAS to no more than 0.12 per 1,000 live births is a stated objective of the national agenda in *Healthy People 2000*. One step toward attaining this is by ensuring that all prospective mothers know what FAS is. Knowledge of FAS was elicited among respondents in two health surveys to determine how much women and men know about the risks of drinking during pregnancy, how knowledge levels have changed over time, and what the implications of these findings are with regard to reducing the level of FAS among newborns.

This Epidemiologic Bulletin examines changes in respondents’ attitudes toward the risks of heavy drinking during pregnancy, their awareness of fetal alcohol syndrome (FAS), and their knowledge of what FAS is. Although the respondents are women and men ages 18 to 44 years, the detailed analyses presented here focus on women only. Data were extracted from the Health Promotion and Disease Prevention (HPDP) supplements of both the 1985 and the 1990 National Health Interview Survey (NHIS).

## Background

One of the important contributions of biomedical research in the past century is the demonstration that personal lifestyle choices make a significant difference in the measure of health and quality of life. Accordingly, a national agenda aimed at considerably reducing preventable death and disability and enhancing quality of life by the year 2000 has been initiated. *Healthy People 2000* (U.S. Department of Health and Human Services 1991) is a substantial effort involving health professionals and citizens, public agencies, and private organizations from all over the United States. Work on the objectives began in 1987 with the creation of a consortium that now includes nearly 300 national membership organizations as well as all State health departments.

More than 300 specific objectives in 22 priority areas were identified in *Healthy People 2000* as goals targeted for achievement by the year 2000. One of these objectives is to reduce the incidence of FAS to no more than 0.12 per 1,000 live births. A step toward attaining this level would be to ensure that all prospective mothers know what FAS is. Although increased knowledge and awareness do not necessarily lead to changes in behavior, it is unlikely that changes such as decreasing or eliminating alcohol consumption during pregnancy will occur in the absence of knowledge of the risks of not doing so. Therefore, three important questions have to be addressed: How much do women in the United States today know about the risks to the fetus that are associated with alcohol consumption during pregnancy, particularly FAS? How have knowledge levels of FAS changed over time? What are the implications of such findings with regard to achieving the *Healthy People 2000* goal for FAS?

The purpose of this study is threefold. First, it will examine changes since 1985 in the percentage of men and women ages 18 to 44 years who believe that heavy drinking during pregnancy increases the chances of miscarriage, mental retardation, low birth weight, and birth defects; who have heard of FAS; and who can correctly describe FAS. Second, it will examine whether differences in these areas of belief, awareness, and knowledge exist among all women, current drinkers, and risk drinkers. Third, it will examine whether differences exist among demographic subgroups in the population of women of childbearing age.

## Methods

### Sources of Data

Data for this study were derived from HPDP supplements in the 1985 and the 1990 NHIS. These questionnaires were first developed to monitor progress on national health objectives for the year 1990 and, later, for the year 2000. In the section involving risks of heavy drinking during pregnancy, awareness of FAS, and knowledge of FAS, survey respondents between the ages of 18 and 44 years were asked a series of questions about their agreement as to whether heavy drinking during pregnancy increases the chances of miscarriage, mental retardation, low birth weight, and birth defects. Heavy drinking was respondent defined; that is, each respondent used his or her own criteria of heavy drinking. Thus, different respondents may have interpreted the questions in many different ways.

Respondents were asked to indicate whether heavy drinking during pregnancy “definitely increases,” “probably increases,” “probably does not increase,” or “definitely does not increase” the chances of these complications. A response of “don’t know” also was valid. The responses “definitely increases” or “probably increases” were collapsed to represent agreement.

These same respondents were then asked if they had ever heard of FAS. Those who replied affirmatively were asked to select a response that best described FAS. The choices were a baby that is “born drunk,” “addicted to alcohol,” or “born with certain birth defects.” (“Born with certain defects” is the correct response.)

### Respondent Categories

Respondents were grouped into several categories, with “all” representing the total U.S. noninstitutionalized population ages 18 to 44 years. “Abstainers” were defined as persons who have had fewer than 12 drinks in any 1 year. “Former drinkers” were defined as persons who have had at least 12 drinks in 1 or more years previously but no drinks in the past year. “Current drinkers” were defined as persons who have had at least 12 drinks per year previously and at least 1 drink in the past year. “Risk drinkers” were defined as persons who drank at levels higher than the levels of moderate drinking suggested in the *Dietary Guidelines for Americans* (USDA/ USDHHS 1990). For women, risk drinking was defined as average alcohol consumption of more than one drink per day. For men, risk drinking was defined as average alcohol consumption of more than two drinks per day.

### Analyses and Tests of Differences

Tests of differences in proportions between response categories in the 1985 and the 1990 HPDP supplements were made by comparing 95 percent confidence intervals;^1^ differences were considered significant when the 95-percent confidence intervals did not overlap. Standard errors used to construct the confidence intervals were based on estimates from SESUDAAN, a computer program that estimates standard errors and takes into account complex sampling designs such as those of the NHIS (Shah 1981). NHIS responses were weighted to be representative of the 1985 and the 1990 U.S. population.

The items selected for analysis from the HPDP supplements fall into dimensions of beliefs, awareness, and knowledge. Responses to items related to risks of heavy drinking during pregnancy essentially represent beliefs or attitudes about heavy drinking. Responses to items related to the respondents having heard of FAS represent awareness, perhaps in large part influenced by public education and the media. Responses to items related to the respondents’ ability to distinguish among three response alternatives and correctly identify FAS as a child born with certain birth defects represent some level of knowledge of FAS.

## Results

### Sample and Overall Findings

Table 1 presents sample sizes, population estimates, and prevalence estimates (or percentage distributions) of all respondents, abstainer, former drinker, current drinker, and risk drinker groups for the population ages 18 to 44 years according to gender. Among this age group, 58 percent of women were current drinkers in 1990, which is significantly different from the 64 percent in 1985; 77 percent of men were current drinkers in 1990, which is significantly different from the 82 percent in 1985. In 1990, risk drinking was characteristic of 10 percent of current drinking women and 12 percent of current drinking men.

Table 2 presents findings among all women and men ages 18 to 44 years in 1985 and 1990 on beliefs about the risks of heavier drinking during pregnancy, their awareness of FAS, and their descriptions of FAS. Data also are presented for abstainers, former drinkers, current drinkers, and risk drinkers in this age group.

The vast majority (89 to 92 percent) of all women in 1990 believed that heavy drinking during pregnancy definitely or probably increases the chances of miscarriage, mental retardation, low birth weight, and birth defects. These beliefs are all significantly higher than those found in 1985 (87 to 88 percent). Also, a significantly higher percentage (73 vs. 62 percent) of women in 1990 had heard of FAS than in 1985. Although 25 percent of women of childbearing age who had heard of FAS in 1985 correctly described FAS as a child born with certain birth defects, 39 percent correctly described FAS in 1990.

Many of the beliefs, awareness, and knowledge increases between 1985 and 1990 were shared among men between the ages of 18 and 44 years, yet a significantly lower percentage of men than women believed in the stated risks of heavier drinking during pregnancy, had heard of FAS, and could correctly describe the syndrome.

For both genders, a larger percentage of current drinkers compared with the general population were aware of the stated risks of heavy drinking and of FAS. Significant differences from the general population in the awareness of FAS also were found among most drinking categories. Abstainers were significantly less likely than the total population to have heard of FAS; former drinkers, current drinkers, and risk drinkers were significantly more likely than abstainers and the general population to have heard of FAS. In being able to describe FAS, however, none of the percentage correct responses among the drinking groups were significantly different from the general population or from the other drinking groups.

### Detailed Differences by Demographic Characteristics

Table 3 presents selected demographic findings on the percentage of women who agree that heavy drinking during pregnancy increases the chances of birth defects. This analysis attempts to determine whether the previously observed 1985 and 1990 increases in beliefs about the risks of birth defects from heavy drinking among all women ages 18 to 44 years also are characteristic of various demographic subgroups of women in this age group. For all women and current drinking women, changes between 1985 and 1990 were fairly consistent and significant across most of the demographic subgroups. The exceptions were the black, Hispanic, less than 12 years of education, less than $20,000 family income, unemployed or not in the labor force, and the divorced/ separated categories, where increases were not significant.

Significant increases also were found among current drinking women compared with the general population of women, because current drinkers were more inclined to agree on the risks of heavier drinking during pregnancy in the first place. However, among women risk drinkers, significant differences across 1985 and 1990 were found only among women 30 to 44 years of age, women with family incomes of $50,000 and over, and women residing in the South. Compared with all women combined, Hispanic women in 1985 and 1990 were less likely to agree that heavy drinking increases the chances of birth defects, as were women with less than 12 years of education.

Table 4 presents the percentage of women in selected demographic subgroups in 1985 and 1990 who reported that they had heard of FAS. A significant increase in the awareness of FAS between 1985 and 1990 was evident among all of the subgroups examined for both all women and current drinking women of childbearing age. However, among subgroups of risk drinkers, significant increases in awareness were found only among groups 30 to 44 years, white, non-Hispanic, employed or other status, married, and living in the Northeast.

Black women, Hispanic women, and women with family incomes of less than $20,000 per year were less likely than all women combined to have heard of FAS. On the other hand, women ages 30 to 44 years, living in the Midwest, and with more than 12 years of education were more likely to have heard of FAS than were all women combined.

Table 5 presents the percentage of all women, current drinkers, and risk drinkers who correctly chose the response that best described FAS, that is, a child born with certain birth defects. Significant increases between 1985 and 1990 in this knowledge generally were found within each of the demographic subgroups examined, except for risk drinkers.

The lack of many significant increases among risk drinkers in their knowledge of FAS between 1985 and 1990 could be attributed to low sample sizes in the subgroups. For risk drinkers, a larger percentage change would be necessary than for the other analytic groups to indicate statistical significance. However, some increases in the correct responses about what FAS is were found among women risk drinkers of childbearing age who were older (ages 30 to 44 years), had family incomes of $20,000 to $34,999, and were divorced or separated.

## Discussion

Increases since 1985 among women ages 18 to 44 years regarding their agreement on the risks of heavy drinking, their awareness of FAS, and their ability to correctly describe FAS are encouraging. The level of knowledge among these women regarding what FAS is, however, is still disturbing. Thirty-nine percent of women of childbearing age *who had heard of FAS* could correctly describe it in 1990. This means that only 29 percent of *all women of childbearing age* (whether they had heard of FAS or not) could correctly describe it. The figure for *all women of childbearing age* in 1985 was only 16 percent. Obviously, more prevention and education efforts are needed to inform women of the dangers of heavy drinking and of any drinking during pregnancy.

The changes between 1985 and 1990 in awareness and knowledge of the dangers of alcohol use and birth defects cannot be attributed to alcoholic beverage warning labels to any great extent, because warning labels were not present on most alcoholic products for sale until 1990 (Hankin et al. 1993) (see the article by Hankin, pp. 62–66). Data in these surveys show that most women of child-bearing age agree that heavy drinking increases the risk of mental retardation and birth defects. However, less than a third can correctly describe FAS. While this may be a contradiction, it seems advisable for prevention to focus on knowledge as opposed to attitudes, where FAS severity may be underestimated. Also, it is problematic whether there is any real relevance to being aware of FAS if most of these women of childbearing age cannot correctly identify what FAS is. Perhaps prevention efforts should focus on the specific birth defects characteristic of FAS.

The number of women and men who believe that FAS is a child born addicted to alcohol may be related to the public attention given to crack babies. Whether such women and men simply believe that a newborn child can “dry out” or recover with abstinence, and thus sustain no lasting harmful effects, is unknown.

Evidence of increased risk of FAS at low levels of alcohol consumption has not been firmly established, but even moderate levels of alcohol intake during pregnancy have been associated with developmental and other problems (Walpole et al. 1990; Waterson and Murray-Lyon 1990; also see the articles by Day and Richardson, pp. 42–48, and Jacobson and Jacobson, pp. 30–36). Since 1983, the American Medical Association has recommended that physicians advise women against any drinking during pregnancy because of the potential dangers of alcohol consumption to the fetus (Waterson and Murray-Lyon 1990).

Those groups who are less likely to believe in the risks of heavier drinking, to be aware of FAS, or to correctly define it may need special targeting for general prevention efforts and specific intervention efforts with women who are pregnant. Results from this study and other studies (Serdula et al. 1991) suggest that such groups often are drinkers who are young (under age 30), black, Hispanic, or with limited years of education—groups already at risk for poor pregnancy outcomes, problems often compounded by socioeconomic status. Weiner and colleagues (1989) suggest that primary prevention efforts and direct interventions that focus on changing drinking behavior have an excellent chance of succeeding, especially when delivered by physicians or other health care providers.

## Figures and Tables

**Table 1:** Sample Sizes, Population Estimates, and Prevalence Estimates of Selected Drinking Categories for Women and Men Ages 18 to 44 Years, 1985 and 1990

	Sample Size		Population Estimate^1^		Prevalence Estimate (%)	
			
Category	1985	1990	1985	1990	1985	1990
Women						
All respondents	10,578	13,055	50,596	53,541	—	—
Abstainer	3,155	4,387	16,063	19,035	32	36
Former drinker	443	768	2,084	3,025	4	6
Current drinker	6,813	7,752	31,951	30,866	64	58
Risk drinker^2^	853	813	3,935	2,929	12	10
Men						
All respondents	8,340	9,973	48,451	51,541	—	—
Abstainer	943	1,402	6,098	8,090	13	16
Former drinker	390	678	2,259	3,520	5	7
Current drinker	6,867	7,760	39,131	39,194	82	77
Risk drinker^2^	1,048	975	5,736	4,702	15	12

1 In thousands. *Abstainer* : fewer than 12 drinks in any 1 year. *Former drinker* : 12 drinks or more a year previously, but no drinks in the past year. *Current drinker* : 12 or more drinks a year previously and at least 1 drink in the past year. *Risk drinker* : average of more than one drink a day (women); average of more than two drinks a day (men).

2 Subgroup of current drinkers.

**Table 2:** Knowledge of Health Risks of Heavy Drinking and Fetal Alcohol Syndrome (FAS), Women and Men Ages 18 to 44 Years, 1985 and 1990

	All (%)		Abstainer (%)		Former Drinker (%)		Current Drinker (%)		Risk Drinker^1^ (%)	
					
Health Risk	1985	1990	1985	1990	1985	1990	1985	1990	1985	1990
Women										
Agree that heavy drinking increases the risk of:										
miscarriage	87	89*	87	88	87	90	89	92*†	87	91
mental retardation	87	90*	86	89*	89	91	88	92*†	87	91
low birth weight	88	91*	88	90*	90	91	90	92*†	88	93*
birth defects	88	92*	87	91*	90	90	89	94*†	88	92
Yes, heard of FAS	62	73*	52†	65*†	67	78*†	67†	77*†	66	76*
FAS description: child born^2^										
drunk	3	4	4	5	3	3	3	4	3	3
addicted to alcohol	72	58*	72	60*	73	54*	71	57*	69	61
with certain birth defects	25	39*	24	36*	24	43*	26	40*	28	36

Men										
Agree that heavy drinking increases the risk of:										
miscarriage	82	82	81	77†	87	82	84	84	80	81
mental retardation	79	81	79	78	87†	81	81	83*	79	80
low birth weight	79	82*	78	79	82	81	80	84*	75	79
birth defects	80	83*	80	80	86†	82	81	85*	79	82
Yes, heard of FAS	49	55*	38†	44†	51	56	50	57*	47	51
FAS description: child born^2^										
drunk	3	4	4	5	3	5	3	4	2	4
addicted to alcohol	73	60*	67	59	78	59*	73	60*	75	64*
with certain birth defects	24	36*	29	37	18	37*	24	36*	23	32

NOTE: Weighted data.

1 Average of more than one drink a day (women); average of more than two drinks a day (men).

2 Excludes respondents who have never heard of FAS.

* Significantly different (*p* < 0.05) from 1985.

† Significantly different (*p* < 0.05) within year from all combined.

**Table 3:** Women Who Agree That Heavy Drinking During Pregnancy Increases Chances of Birth Defects According to Selected Demographic Characteristics, 1985 and 1990

	All (%)		Current Drinker^1^ (%)		Risk Drinker^2^ (%)	
			
Category	1985	1990	1985	1990	1985	1990
All Combined	88	92*	89	94*	88	92
Age						
18–29 years	91†	93*	93†	96*†	93†	94
30–44 years	85†	90*	85†	93*	81†	90*
Race						
White	88	92*	90	94*	88	93
Black	88	90	88	93*	85	85
Hispanic^3^						
Yes	81†	85†	86	92	91	97
No	88	92*	90	94*	88	92
Education						
Less than 12 years	83†	86†	86	92*	86	91
12 years	88	92*	89	94*	89	90
More than 12 years	89	93*†	90	95*	87	94
Family Income						
Less than $20,000	89	90	91	94*	90	94
$20,000 to $34,999	88	93*	90	95*	91	93
$35,000 to $49,999	88	93*	89	94*	90	93
$50,000 and over	87	93*	87	94*	77	93*
Employment Status						
Employed	88	92*	89	94*	88	91
Other^4^	88	90	90	94*	87	94
Marital Status						
Married	88	92*	89	94*	86	91
Divorced/separated	86	91*	87	93	86	90
Never married	89	92*	91	95*	91	95
Region						
Northeast	87	92*	89	94*	87	92
Midwest	90†	93*	91	94*	90	92
South	87	92*	88	95*	84	94*
West	86	89*†	89	93*	90	90

NOTE: Weighted data.

1 12 or more drinks a year previously and at least 1 drink in the past year.

2 Average of more than 1 drink per day.

3 Not mutually exclusive from race categories.

4 Other includes unemployed and not in the labor force.

* Significantly (*p* < 0.05) different from 1985.

† Significantly (*p* < 0.05) different within year from all combined.

**Table 4:** Women Who Have Heard of Fetal Alcohol Syndrome According to Selected Demographic Characteristics, 1985 and 1990

	All (%)		Current Drinker^1^ (%)		Risk Drinker^2^ (%)	
			
Category	1985	1990	1985	1990	1985	1990
All Combined	62	73*	67	77*	66	76*
Age						
18–29 years	60	69*†	65	74*†	64	73
30–44 years	64	76*†	69	80*†	69	79*
Race						
White	65†	76*†	69	79*	67	78*
Black	48†	59*†	52†	61*†	53	54†
Hispanic^3^						
Yes	37†	52*†	45†	62*†	43†	67
No	64	75*†	68	78*	67	76*
Education						
Less than 12 years	39†	53*†	46†	60*†	50†	60†
12 years	58†	69*†	62†	72*†	61	73
More than 12 years	75†	83*†	77†	85*†	77†	82
Family Income						
Less than $20,000	56†	63*†	62†	70*†	63	68
$20,000 to $34,999	65	73*	69	76*	67	75
$35,000 to $49,999	70†	78*†	72†	81*	66	78
$50,000 and over	71†	82*†	72	84*†	73	86†
Employment Status						
Employed	64	74*	68	78*	70	78*
Other^4^	59	70*†	66	76*	55	70*
Marital Status						
Married	65	76*†	69	80*	68	79*
Divorced/separated	60	71*	65	75*	64	75
Never married	55†	67*†	62†	72*†	64	73
Region						
Northeast	61	70*	66	76*	60	77*
Midwest	67†	79*†	70	82*†	69	78
South	59	72*	66	76*	66	76
West	62	71*	66	75*	67	73

NOTE: Weighted data.

1 12 or more drinks a year previously and at least 1 drink in the past year.

2 Average of more than 1 drink per day.

3 Not mutually exclusive from race categories.

4 Other includes unemployed and not in the labor force.

* Significantly (*p* < 0.05) different from 1985.

† Significantly (*p* < 0.05) different within year from all combined.

**Table 5:** Women Who Correctly Describe Fetal Alcohol Syndrome According to Selected Demographic Characteristics, 1985 and 1990

	All (%)		Current Drinker^1^ (%)		Risk Drinker^2^ (%)	
			
Category	1985	1990	1985	1990	1985	1990
All Combined	25	39*	26	40*	28	36
Age						
18–29 years	26	37*	26	38*	30	30
30–44 years	24	40*	25	41*	25	41*
Race						
White	25	39*	26	40*	27	37
Black	26	33*†	23	31†	26	‡
Hispanic^3^						
Yes	33†	39	30	35	‡	‡
No	25	39*	26	40*	27	36
Education						
Less than 12 years	28	35	25	31†	‡	41
12 years	22	34*†	23	34*†	24	30
More than 12 years	27	43*†	28	45*†	33	39
Family Income						
Less than $20,000	25	35*†	26	34*†	31	31
$20,000 to $34,999	24	37*	24	40*	20	43*
$35,000 to $49,999	24	39*	26	41*	25	39
$50,000 and over	25	43*	25	43*	36	33
Employment Status						
Employed	24	38*	25	39*	28	33
Other^3^	27	40*	29	43*	27	43
Marital Status						
Married	25	39*	27	41*	27	41
Divorced/separated	22	37*	20†	36*	17	34*
Never married	26	38*	26	38*	32	28
Region						
Northeast	25	39*	27	41*	29	30
Midwest	23	35*	24	37*	27	32
South	26	37*	26	37*	28	36
West	26	45*†	28	46*†	27	45

NOTE: Weighted data. Excludes respondents who have never heard of fetal alcohol syndrome.

1 12 or more drinks a year previously and at least 1 drink in the past year.

2 Average of more than 1 drink per day.

3 Not mutually exclusive from race categories.

4 Other includes unemployed and not in the labor force.

* Significantly (*p* < 0.05) different from 1985.

† Significantly (*p* < 0.05) different within year from all combined.

‡ Fewer than 15 respondents.

## Footnotes

1 A confidence interval is a range of values that carries a specified probability of including a parameter, such as a percentage estimate. With a 95-percent confidence interval, one is confident that—95 times out of 100—the “true” value will be within the range of values. When the 95-percent confidence intervals of two like measures do not overlap, the measures are considered to be significantly (*p* < 0.05) different.
